# Factors affecting influenza vaccination in adults aged 50-64 years with high-risk chronic diseases in South Korea

**DOI:** 10.1080/21645515.2018.1556075

**Published:** 2019-01-16

**Authors:** Hyeongap Jang, Joon Hyung Kim

**Affiliations:** a GSK, Seoul, South Korea; b GSK, Rockville, MD, USA

**Keywords:** vaccination, influenza, South Korea, knowledge, attitude

## Abstract

Influenza is a communicable disease with most of the mortality burden falling on high-risk populations and those with pre-existing comorbidities and chronic diseases. In South Korea, adults aged 50–64 years are recommended for influenza vaccination, but no government financial support is offered to encourage vaccination uptake, which has led to suboptimal vaccination rates and significant public health concerns. The purpose of this study was to identify the factors affecting influenza vaccine uptake in adults aged 50–64 years and to compare high-risk and non-high-risk groups. We conducted randomized telephone questionnaires in South Korea on influenza vaccination-related behavioural factors in adults aged 50–64 years based on their vaccination history during the 2015–2016 flu season. The vaccination rate was 29.9% in non-high-risk adults aged 50–64 years and 41.3% in high-risk adults aged 50–64 years, which is considerably lower than the 81.7% rate in adults aged ≥65 years. Individuals who reported awareness of the potential severity of influenza, the importance and safety of vaccination, and who had experienced influenza after immunization or received a healthcare recommendation reported higher influenza vaccination rates. Therefore, highlighting awareness of influenza disease and vaccination through public campaigns and by information from healthcare professionals could represent opportunities to improve vaccination uptake in this population.

## Introduction

Influenza is a communicable disease that occurs in yearly seasonal epidemics due to its viral characteristics, including constant changes to the influenza virus genome.^1^ The global influenza burden is estimated at 290,000 to 650,000 deaths, and 3,000,000 to 5,000,000 cases of severe illness per year, with the majority occurring in high-risk populations.^2^ Therefore, influenza vaccination is recommended in high-risk individuals and for those who are likely to have contact with high-risk individuals.^2^

According to the World Health Organization (WHO), the United States (US) Centers for Disease Control and Prevention (CDC) and the European (EU) CDC, the ≥65 years age-group is defined as high-risk.^2–4^ Influenza vaccination is also recommended for individuals aged 50–64 years by the South Korean CDC^5^ although this age demographic is not classified as high-risk. Thus, the South Korea government supports vaccination costs for individuals aged ≥65 years, but not for those aged 50–64 years. In 2015, the vaccination rate in the 50–64 years age-group was 31.4%, which is much lower than the 81.7% reported in the age group ≥65 years,^6,7^ which is cause for significant public health concern.

Comorbidities and chronic diseases can frequently occur in this age-group, which increase the chances of more severe illness or mortality during an influenza infection.^8^ Considering this suboptimal vaccination rate, studying behaviours around influenza vaccination in 50–64 years age-group is important for understanding the barriers to immunization, and how to increase the vaccination uptake in this specific population that does not opt for vaccination.

There are currently no behavioural vaccination studies available from the private market for the 50–64 years age-group,^9–12^ and existing studies involving 50–64 years age-groups do not account for comparisons with individuals aged ≥65 years. Considering the vast differences of influenza vaccination coverage and market characteristics between reimbursed groups (≥65 years) and non-reimbursed groups (50–64 years), focused studies on non-reimbursed populations need to be conducted. It is also important to compare vaccination behaviour of high-risk chronic disease groups with non-high-risk groups in this age group. Finally, there is a need to gain an understanding of how to engage with these population groups to improve vaccine uptake.

**Figure 1.**
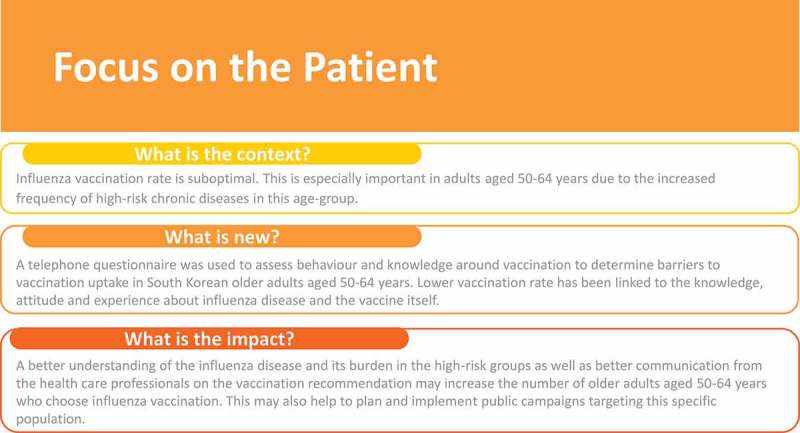
Focus on the patient.

The purpose of this study was to survey adults aged 50–64 years in South Korea (including high vs non-high-risk group comparisons) who are not covered by a national immunization programme to identify factors affecting influenza vaccine uptake (Figure 1).

## Results

The overall response rate of the telephone questionnaire was 47.9%. A total of 1,675 individuals completed interview questionnaires. Fourteen samples were excluded due to poor response quality. In total, 1,661 samples were analyzed in this study.

## Descriptive analysis: individual socioeconomic factors

### Gender and age

The overall vaccination rate of adults aged 50–64 years was 31.9% (530 of 1,661 study participants) during the 2015–2016 flu season. The male vaccination rate (26.7% of 828 men) was significantly lower than the female vaccination rate (37.1% of 833 women) with a crude odds ratio (cOR) for vaccinated female of 1.62 (*P* = < 0.01). The vaccination uptake rates for individuals aged 50–54, 55–59, and 60–64 years were 26.7%, 31.0%, and 41.1% respectively. Individuals aged 60–64 years reported a significantly higher vaccination rate than the 50–54 years age-group (cOR = 1.92, *P* = < 0.01) (Table 1).

**Table 1.** Baseline characteristics and cOR on vaccination of the study subjects.

	Strata	No. of respondent	No. vaccinated	Vaccination rate (%*)	cOR	95% CI	p-value
Total		1661	530	31.9			
Gender	Male	828	221	26.7	1.00	Reference	
	Female	833	309	37.1	1.62	1.31–2.00	<0.01
Age	50–54	644	172	26.7	1.00	Reference	
	55–59	594	184	31.0	1.23	0.96–1.58	0.10
	60–64	423	174	41.1	1.92	1.48–2.49	<0.01
Education	≤High school	1079	398	36.9	1.00	Reference	
	College or University	500	115	23.0	0.51	0.40–0.65	<0.01
	≥Graduate school	82	17	20.7	0.45	0.26–0.77	<0.01
Employment status	Housewife	349	118	33.8	1.00	Reference	
	Employed	490	196	40.0	1.31	0.98–1.74	0.07
	Others	822	216	26.3	0.70	0.53–0.91	0.01
Average Household Income (x 10^3^ KRW/month)	≤3000	816	299	36.6	1.00	Reference	
	3001–5000	508	129	25.4	0.59	0.46–0.75	<0.01
	≥5001	337	102	30.3	0.75	0.57–0.99	0.04
Region	Metropolitan city	762	247	32.4	1.00	Reference	
	Others	899	283	31.5	0.96	0.78–1.18	0.68
Cohabitant	No	103	39	37.9	1.00	Reference	
	Yes	1558	491	31.5	0.76	0.50–1.14	0.18
High-risk cohabitant for flu	No	1243	377	30.3	1.00	Reference	
	Yes	418	153	36.6	1.33	1.05–1.67	0.02
Subjective health status	Good	1204	348	28.9	1.00	Reference	
	Not good	457	182	39.8	1.63	1.30–2.04	<0.01
High-risk chronic disease^†^	None	1363	407	29.9	1.00	Reference	
	Yes	298	123	41.3	1.65	1.28–2.14	<0.01
	Cardiovascular	75	21	28.0	0.91	0.54–1.53	0.73
	Endocrine	170	76	44.7	1.90	1.37–2.63	<0.01
	Respiratory	28	11	39.3	1.52	0.71–3.27	0.29
	Renal/Hepatic/Immunodeficiency/Neurologic	49	26	53.1	2.66	1.50–4.71	<0.01

*calculated as the percentage of vaccinated individuals over the number of the total number of individuals in each respective category, therefore % = (number of vaccinated/number of respondent in each category)x100; ^†^ The sum of numbers of the chronic disease group can exceed total chronic group due to patients with multiple chronic diseases

cOR, crude odds ratio; No., number; CI, confidence interval; KRW, Korean Won.

### Education, income and cohabitants

More highly educated individuals reported a significantly lower vaccination rate. The overall vaccine uptake rate in individuals who had graduated from high school or a lower level of educational institution was 36.9%. In comparison, the vaccination rate in individuals who had finished graduate school was only 20.7%, with a cOR of 0.45 *(P = < *0.01). Individuals who had graduated from college or university also reported a lower vaccination rate of 23.0% with a cOR of 0.51 (*P = *< 0.01).

Similarly, a higher income was negatively associated with vaccination uptake rate. Individuals with a middle- or high-level income showed a lower rate of vaccination (25.4% and 30.3%, respectively) than people with a lower income (36.6%). These differences were also statistically significant, with a cOR of 0.59 (*P* = < 0.01) and 0.75 (*P* = 0.04), respectively (Table 1). In total, 418 people were cohabitating with individuals considered at high-risk for influenza, and their vaccination rate was 36.6%, compared with a vaccination rate of 30.3% in people cohabitating with individuals not considered to be at high risk (cOR 1.33, *P* = 0.02) (Table 1).

### Health status

The vaccination rate in 298 high-risk patients with chronic diseases was 41.3%, which was significantly higher compared to the non-high-risk group (29.9%, cOR = 1.65, *P* = < 0.01) (Table 1). Only the endocrine disease group (including diabetes) (44.7%) and the other chronic disease group (53.1%) showed significantly higher vaccination rates. The cORs for both groups were 1.90 (*P = *< 0.01) and 2.66 *(P = < *0.01), respectively. The cardiovascular disease group had a numerically lower vaccination rate than the non-high-risk group (28.0%, cOR 0.91, *P* = 0.73), although this difference was not statistically significant.

## Descriptive analysis: KAP (knowledge, attitude, and practice) questionnaires

### Knowledge

In total, 453 (27.3%) respondents were unaware of differences between the symptoms of influenza infection and those of the common cold, and 700 (42.1%) respondents were unaware of the importance of annual influenza vaccination. Both factors had a negative impact on vaccination rates with cOR = 0.78 (*P* = 0.04) and cOR = 0.11 (*P = *< 0.01) respectively. A total of 276 (16.6%) respondents were not aware that influenza vaccination reduced the severity of influenza during infection (cOR = 0.55, *P* = < 0.01), and 669 (40.3%) respondents reported an awareness of the risk of experiencing side-effects following influenza vaccination (cOR = 1.29, *P* = 0.02) (Table 2).

**Table 2.** Descriptive analysis and cOR for KAP questionnaire.

Questionnaire items	Strata	Respondents		Vaccination rate (%^†^)	cOR	95% CI	p-value
		No	%*				
***Knowledge***							
Influenza and colds are different diseases and symptoms also differ	Know	1208	72.7	33.4	1.00	Reference	
	I don’t know	453	27.3	28.0	0.78	0.61–0.99	0.04
Influenza viruses change continuously, and we can get influenza regardless of previous influenza experience	Know	1481	89.2	32.0	1.00	Reference	
	I don’t know	180	10.8	31.1	0.96	0.69–1.34	0.81
We need to get an influenza vaccine every year	Know	961	57.9	48.5	1.00	Reference	
	I don’t know	700	42.1	9.1	0.11	0.08–0.14	<0.01
We can get influenza even after influenza vaccination	Know	1435	86.4	31.8	1.00	Reference	
	I don’t know	226	13.6	32.3	1.02	0.76–1.38	0.89
Influenza vaccination reduces influenza severity when we have influenza	Know	1385	83.4	33.9	1.00	Reference	
	I don’t know	276	16.6	22.1	0.55	0.41–0.75	<0.01
Influenza vaccination has a risk of side-effects	Know	992	59.7	29.6	1.00	Reference	
	I don’t know	669	40.3	35.3	1.29	1.05–1.59	0.02
***Attitude (Belief)***							
I think influenza is a serious disease	Agree	911	54.8	38.5	1.00	Reference	
	Disagree	750	45.2	23.9	0.50	0.40–0.62	<0.01
I think influenza vaccination reduces influenza significantly	Agree	1419	85.4	34.6	1.00	Reference	
	Disagree	242	14.6	16.1	0.36	0.25–0.52	<0.01
I have concerns on vaccination side-effects and I think influenza vaccination is not safe	Agree	488	29.4	26.0	1.00	Reference	
	Disagree	1173	70.6	34.4	1.49	1.18–1.88	<0.01
The cost of influenza vaccination is a burden for me	Agree	889	53.5	32.2	1.00	Reference	
	Disagree	772	46.5	31.6	0.97	0.79–1.20	0.81
***Experience***							
I have experienced influenza before	Yes	417	25.1	38.6	1.00	Reference	
	No	1244	74.9	29.7	0.67	0.53–0.85	<0.01
I have experienced influenza after influenza vaccination	Yes	238	14.3	51.7	1.00	Reference	
	No	1423	85.7	28.6	0.37	0.28–0.50	<0.01
I received an influenza vaccination recommendation from a healthcare provider	Yes	470	28.3	36.6	1.00	Reference	
	No	1191	71.7	30.1	0.74	0.59–0.93	0.01
One of my family members has experienced influenza	Yes	433	26.1	31.9	1.00	Reference	
	No	1228	73.9	31.9	1.00	0.79–1.27	0.98

*calculated as the percentage of respondents in each strata over the total responses received by respective item (equals 1661), therefore % = (number of respondents in each strata/number of respondents in both strata of the respective item)x100

^†^calculated as the percentage of vaccinated individuals in each strata of the respective item, therefore % = (number of vaccinated individuals/item’s strata population)x100

cOR, crude odds ratio; No., number; CI, confidence interval; KAP, knowledge, attitude (belief), and practice experience.

### Attitude

A total of 750 (45.2%) respondents did not agree with the statement that influenza was a serious disease, and reported a much lower vaccination rate (cOR = 0.50, *P* = < 0.01). A total of 242 respondents (14.6%) disagreed with the benefit of vaccination and showed a strong negative correlation with vaccination (cOR = 0.36, *P* = < 0.01) (Table 2).

### Practice

In total, 1,244 (74.9%) respondents had not previously experienced influenza disease and 238 (14.3%) respondents had influenza disease after vaccination. Both of these factors resulted in a significantly higher vaccine uptake compared to other respondents, cOR = 0.67 (*P* = < 0.01) and cOR = 0.37 (*P* = < 0.01) respectively. A total of 1,191 (71.7%) respondents who were not recommended the influenza vaccination from a healthcare professional (HCP) showed a lower vaccination rate (cOR = 0.74, *P* = 0.01) (Table 2).

## Regression model: individual socioeconomic factors

We compared the results of socioeconomic factors between non-high-risk and high-risk groups (Table 3).

**Table 3.** Regression result for influenza vaccination.

		Non-high-risk group			High-risk group		
		aOR	95% CI	p-value	aOR	95% CI	p-value
Gender	Male	1.00	Reference		1.00	Reference	
	Female	1.17	0.81–1.68	0.40	1.43	0.63–3.24	0.39
Age	Per one year	1.05	1.01–1.08	0.01	1.04	0.96–1.12	0.33
Education	≤High school	1.00	Reference		1.00	Reference	
	College or University	0.63	0.44–0.88	0.01	0.45	0.19–1.03	0.06
	≥Graduate school	0.45	0.23–0.90	0.02	0.06	0.01–0.37	<0.01
Employment status	Housewife	1.00	Reference		1.00	Reference	
	Employed	0.86	0.54–1.37	0.53	2.34	0.87–6.27	0.09
	Others	0.73	0.50–1.06	0.10	1.82	0.83–3.96	0.13
Average Household Income (x 10^3^ KRW/month)	≤3000	1.00	Reference		1.00	Reference	
	3001–5000	1.01	0.72–1.43	0.94	0.57	0.26–1.24	0.15
	≥5001	1.52	1.00–2.30	0.05	0.90	0.33–2.48	0.84
Region	Metropolitan city	1.00	Reference		1.00	Reference	
	Others	0.99	0.76–1.30	0.95	0.56	0.30–1.06	0.08
Cohabitant	No	1.00	Reference		1.00	Reference	
	Yes	0.82	0.44–1.52	0.53	1.32	0.45–3.87	0.61
High-risk cohabitant for influenza	No	1.00	Reference		1.00	Reference	
	Yes	1.19	0.87–1.63	0.28	0.69	0.34–1.38	0.29
Subjective health status	Good	1.00	Reference		1.00	Reference	
	Not good	1.13	0.82–1.56	0.46	2.45	1.29–4.65	0.01
***Knowledge***							
Influenza and colds are different diseases and symptoms also differ	I don’t know	1.00	Reference		1.00	Reference	
	Know	1.25	0.91–1.71	0.17	2.28	1.13–4.59	0.02
Influenza viruses change continuously, and we can get influenza regardless of previous influenza experience	I don’t know	1.00	Reference		1.00	Reference	
	Know	0.90	0.57–1.42	0.65	1.23	0.44–3.45	0.69
We need to get an influenza vaccine every year	I don’t know	1.00	Reference		1.00	Reference	
	Know	8.96	6.35–12.66	<0.01	4.42	2.16–9.06	<0.01
We can get influenza even after influenza vaccination	I don’t know	1.00	Reference		1.00	Reference	
	Know	0.86	0.57–1.32	0.50	0.92	0.41–2.06	0.83
Influenza vaccination reduces influenza severity when we have influenza	I don’t know	1.00	Reference		1.00	Reference	
	Know	0.92	0.61–1.39	0.70	1.28	0.48–3.43	0.62
Side-effects can occur after influenza vaccination	I don’t know	1.00	Reference		1.00	Reference	
	Know	1.10	0.83–1.47	0.51	0.56	0.31–1.05	0.07
***Attitude (Belief)***							
I think influenza is a serious disease	Disagree	1.00	Reference		1.00	Reference	
	Agree	1.49	1.13–1.96	0.01	1.92	0.97–3.79	0.06
I think influenza vaccination reduces influenza significantly	Disagree	1.00	Reference		1.00	Reference	
	Agree	1.34	0.84–2.13	0.22	6.35	1.47–27.51	0.01
I have concerns about vaccine side-effects and I think the influenza vaccine is not safe	Disagree	1.00	Reference		1.00	Reference	
	Agree	0.91	0.67–1.24	0.55	0.21	0.10–0.45	<0.01
The cost of the influenza vaccine is a burden for me	Disagree	1.00	Reference		1.00	Reference	
	Agree	0.86	0.65–1.14	0.29	0.66	0.35–1.24	0.20
***Experience***							
I have experienced influenza before	No	1.00	Reference		1.00	Reference	
	Yes	0.97	0.65–1.46	0.90	0.90	0.39–2.11	0.81
I experienced influenza after vaccination	No	1.00	Reference		1.00	Reference	
	Yes	3.09	1.96–4.87	<0.01	2.03	0.75–5.53	0.17
I received an influenza vaccination recommendation from a healthcare provider	No	1.00	Reference		1.00	Reference	
	Yes	0.98	0.72–1.32	0.89	2.04	1.09–3.84	0.03
One of my family members has experienced influenza before	No	1.00	Reference		1.00	Reference	
	Yes	0.83	0.57–1.19	0.31	1.00	0.46–2.16	1.00

aOR, adjusted odds ratio; CI, confidence interval; KRW, Korean Won.

### Socioeconomic factors

In the non-high-risk group, age, education level, and income were significantly correlated with vaccination rate. An increase in age by one year was significantly associated with higher rate of vaccination (adjusted odds ratio [aOR] = 1.05, *P* = 0.01). Individuals who had graduated from college or university (aOR = 0.63, *P* = 0.01) and those who had finished graduate school (aOR = 0.45, *P* = 0.02) had a significantly lower aOR when compared to individuals who had graduated from high school or a lower level educational institute. A high-income level was positively associated with vaccination: individuals who earned ≥5,000,001 Korean Won (KRW) had a high aOR (1.52, *P* = 0.05) compared with those who earned ≤3,000,000 KRW. In the high-risk group, education level was the only significant socioeconomic variable correlated with the influenza vaccination rate. Individuals who had finished graduate school were less likely to be vaccinated (aOR = 0.06, *P* = < 0.01) when compared to those who had graduated from high school or a lower level educational institute. Individuals who had graduated from college or university also showed a low aOR (0.45), but only a marginal confidence interval (CI) (*P* = 0.06). A poorer subjective health status was also a significant factor for a higher vaccination uptake in the high-risk group (aOR = 2.45, *P* = 0.01). This is in contrast to the lower vaccination uptake in the non-high-risk respondents (aOR = 1.13, *P* = 0.46) (Table 3).

## Regression model: KAP questionnaires

We compared the results of the KAP (knowledge, attitude (belief), and practice) questionnaire between non-high-risk and high-risk groups (Table 3).

### Knowledge

Individuals who reported awareness of the differences between the symptoms of an influenza infection and the common cold showed a significant correlation with the rate of vaccination uptake in the high-risk group (aOR = 2.28, *P* = 0.02) but not in the non-high-risk group. Individuals who were aware of the need for annual vaccination showed a strong correlation with vaccination in both the non-high-risk (aOR = 8.96, *P* = < 0.01) and the high-risk (aOR = 4.42, *P* = < 0.01) group.

### Attitude

The belief that influenza is a serious disease showed a positive correlation with vaccination uptake in the non-high-risk group (aOR = 1.49, *P* = 0.01), but was marginal in the high-risk group (aOR = 1.92, *P* = 0.06). A belief that influenza vaccination significantly reduced influenza disease was strongly correlated with vaccination uptake in the high-risk group (aOR = 6.35, *P* = 0.01), but was not significant in the non-high-risk group. The high-risk group also showed that a negative belief in the risk of side-effects after vaccination had a strong negative correlation with vaccination (aOR = 0.21, *P* = < 0.01). The cost burden was not significant in either group.

### Practice

People who had previously experienced influenza after vaccination showed a positive correlation with influenza vaccination in the non-high-risk group (aOR = 3.09, *P* = < 0.01). Individuals in the high-risk group, who were recommended influenza vaccination by a HCP, showed a positive correlation with vaccination (aOR = 2.04, *P* = 0.03) while this was not the case in the non-high-risk group.

## Discussion

Suboptimal vaccination rates in adults aged 50–64 years, especially in individuals with high-risk chronic diseases, is one of the main public health concerns in South Korea. We conducted a nationwide questionnaire survey in adults aged 50–64 years who were not financially motivated by the government to participate in an influenza vaccination programme, to document individuals who did not receive vaccinations and the possible reasons why. We compared descriptive analyses and regression analyses of the high-risk versus non-high-risk chronic disease groups to generate hypotheses on how to increase influenza vaccine uptake in the community.

Based on socioeconomic variables, our analysis showed that female respondents were more likely to receive influenza vaccination, but this was not evident in our multivariate analysis. This is in line with previous studies demonstrating inconsistent evidence regarding gender as a factor in influenza immunization uptake.^9,11,12^ However, studies from other Asian countries, including South Korea, Hong Kong and Singapore, showed higher vaccination rates in women than in men.^13–16^ Husaini *et al*. found that women used health care services more often than men and were also more likely to follow preventive healthcare recommendations.^17^

Age was significantly associated with influenza vaccination in our bivariate analysis, but it was only significant in the non-high-risk group in our multivariate analysis. Interestingly, many previous studies have also reported a positive association between increasing age and influenza vaccination uptake.^9,11,12,14–16,18,19^

In this study, a higher level of education was associated with a lower vaccination rate. This result was supported by previous studies in South Korea which showed similar results following bivariate analyses.^14,19^ However, the majority of studies, which included education level as a variable in multivariate analyses did not show significant correlations with influenza vaccination.^9,11,14,15,18,19^

Income was not a significant factor for influenza vaccination in this study, and many other studies supported these findings based on inconsistent results.^9,11,15,16,18,19^ However, Lee *et al*. reported that individuals aged <50 years demonstrated positive correlations between income and vaccination, while individuals aged ≥50 years showed an inverse correlation.^14^

The region where the participants lived had no association with vaccination rates in our study, however, in two previous studies in South Korea, it was reported that rural areas had significantly higher vaccination rates than urban regions.^14,15^ A potential reason for these differing results may be that previous studies included individuals aged ≥65 years in the study population who usually have different price sensitivity and accessibility to public health centres by region. Furthermore, nation-wide, free vaccination programmes were only provided in private health care centres after 2015, potentially causing biases between socioeconomic factors such as income and region. However, the influences of geographic region and income levels need to be further investigated in future studies.

Subjective health status was not a significant factor in non-high-risk respondents, but poor subjective health status was associated with a higher vaccination rate in the high-risk group, which has also been reported in other studies.^9,10,16,19,20^

In KAP variables, awareness of symptomatic differences between influenza disease and the common cold was a significant factor in the high-risk group. Schmid *et al*. commented that lack of knowledge of influenza disease and vaccination acted as a barrier against influenza vaccination.^12^ Our study showed a robust correlation between individuals understanding the importance of annual vaccination and vaccine uptake. This finding was supported by several studies that also reported that this knowledge was a strong factor in vaccination uptake.^10,11,13,19^

Regarding attitude, agreeing that influenza was a serious disease was significantly associated with higher vaccination rates in the non-high-risk group but not in the high-risk group. Trust in vaccine efficacy and concerns regarding vaccine safety were significantly associated with vaccine uptake in high-risk individuals, which was also supported by the majority of related studies.^9,10,18,20^

In the non-high-risk group, individuals who had experienced influenza after vaccination during the previous flu seasons reported a significantly higher vaccination rate than individuals who had not experienced influenza after vaccination. To the best of our knowledge, no similar study assessing this factor has been carried out. As a large majority of HCPs have concerns that previous “vaccine failure” experiences, regardless of whether this is true or not, may cause distrust in vaccination, we believe that our findings need to be confirmed by further studies to alleviate such concerns. Recommendations by HCPs were positively associated with vaccination in high-risk groups in this study. These results are in line with several other studies where HCP referral was strongly associated with influenza vaccination uptake.^4,9,10,13^

This study utilized a large scale, nationwide, cross-sectional questionnaire with stratified randomized sampling to investigate various socioeconomical and behavioural factors influencing influenza vaccination. The strength of this study is the representativeness of sampling and the comprehensive questionnaire which includes vaccination-related KAP questions.

However, this study does have some limitations: 1) probable information bias, 2) probable confounders, from the observational study design, and 3) reverse causality, the nature of cross-sectional study design. To minimize these limitations, conducting the questionnaire prior to the influenza vaccination season and the prospective collection of an accurate vaccination history from medical records of each enrolled patient rather than depending on self-reporting would be needed in future studies. Besides, this study focused on high-risk comorbidities to determine the confounding effects of this variable on other independent variables. Further investigations on meaningful confounders such as the influenza experience post influenza vaccination or the effects of the educational level would make interesting topics for future studies.

## Conclusion

Our study showed suboptimal vaccination rates in high-risk adults aged 50–64 years. Vaccination rates in high-risk individuals aged 50–64 years were higher than for non-high-risk adults of same age, but much lower than vaccination rates in adults aged ≥65 years. Vaccination rates in patients with a chronic cardiovascular disease were numerically lower than in the non-high-risk group. Our study provides insights into potential public-campaign targets and messages. To increase vaccination coverage in high-risk 50–64-year-olds, we need further conclusive studies on individuals with a higher education level and in people who perceive their health conditions positively.

The public message also needs to be further investigated to highlight the differences between influenza disease and the common cold, and the importance of annual influenza vaccination. Promoting positive public perception of vaccine effectiveness and reassuring people about the safety of influenza vaccination are also important for future studies. Lastly, HCP recommendations for vaccination would be helpful for adults aged 50–64 years classified as high-risk, and further studies are needed to increase the uptake of for influenza vaccination in this age demographic.

## Methods

### Study population and sampling

In 2016, stratified random telephone questionnaires were conducted nationwide in South Korea on influenza vaccination-related knowledge, attitudes, and behavioural practices in adults aged 50–64 years. Random sampling was stratified by age, sex, and region. The questionnaire was conducted by Nielson Korea; a company specialized in computer-assisted telephone interviewing (CATI) systems. Telephone numbers were generated by random digit dialling (RDD) for landline, mobile, and Internet telephone numbers.

### Conceptual model

**Figure 2.**
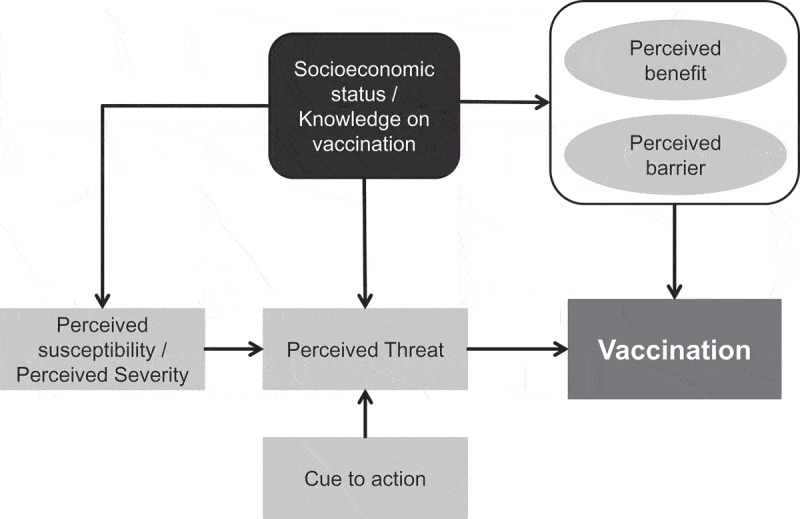
Health belief model: Conceptual framework for analysis. The arrows indicate the direction of the exerted effect. For example, the decision to vaccinate is determined by the perceptions that a person has with respect to the a) benefit of the vaccine such as its effectiveness (‘perceived benefit’), b) risk or barriers to vaccination such as potential side effects (‘perceived barrier’), and c) disease severity (‘perceived threat’). These determinants of vaccination are further influenced by other variables such as: the ‘cue to action’ or else a physician’s recommendation, the socioeconomic status of the person, the knowledge about vaccinations and the perceived susceptibility to the disease influencing the ‘perceived threat’ determinant; the determinants of ‘perceived benefit/barrier’ are influenced by the socioeconomic and knowledge status of each person.^18,23^

The study outcome for our health-belief model was the influenza vaccination history during the 2015–2016 season, identifying vaccination rate, specifically in 50–64-year-olds, as the dependent variable. We identified independent variables based on a health-belief model used widely for behavioural vaccination studies. We selected questions following a literature review for behavioural vaccination studies.^4,13–16,18–22^ Based on the health-belief model and literature review, we identified seven factors that can also be defined as the model’s independent variables 1) perceived susceptibility, 2) perceived severity, 3) perceived benefits, 4) perceived barriers, 5) cues to action, 6) socioeconomic factors and, 7) knowledge of vaccination (Figure 2).^18,23^ Each factor included two to five questions on knowledge, attitude (belief), and practice (KAP) experience. During the interview, we grouped questions based on KAP rather than health-belief categories since KAP questionnaires were more comprehensible for the interviewees.

### Definitions of independent variables

*Individual socioeconomic factors* included age, sex, education level, monthly income, and employment status. Age-groups were categorized as: 1) 50–54, 2) 55–59 and, 3) 60–64 years; education level as: 1) high school graduation or less, 2) college or university, and 3) graduated school or more; monthly income as: 1) ≤3,000,000 KRW which is approximately 2,600 US dollar (USD) (1 USD = 1,160.5 KRW in 2016^24^), 2) 3,000,001–5,000,000 KRW, and 3) ≥5,000,001 KRW which is approximately 4,300 USD; and employment status as: 1) housewife, 2) employed, and 3) other such as student or unemployed.

*Perceived susceptibility factors* included three variables: 1) subjective health status, 2) high-risk disease group and 3) experience of influenza-related illness. We grouped seven disease-related groups into four groups based on the number of patients with: 1) chronic cardiovascular disease, 2) chronic endocrine disease including diabetes, 3) chronic respiratory disease, and 4) other (including chronic hepatic, renal, immunodeficiency and neurologic disease).

*Perceived severity factors* included three variables: 1) knowledge of the difference between a cold and influenza, 2) knowledge of the possibility of re-infection, and 3) the belief that influenza is a severe disease.

*Perceived benefit factors* included five variables: 1) the existence of cohabitant, 2) the existence of high-risk cohabitant for influenza, 3) knowledge of vaccination reducing the severity of influenza, 4) belief that influenza vaccination significantly reduced the risk of infection, and 5) familial experience with influenza.

*Perceived barrier factors* included five variables: 1) knowledge of the possibility of influenza infection after vaccination, 2) knowledge of the possibility of side-effects after vaccination, 3) belief that influenza vaccination is not safe, 4) perception that influenza vaccination is expensive, and 5) experience with influenza infection after vaccination.

*Cue to action factors and Knowledge of vaccination* included one variable each: the recommendation from a HCP and the knowledge that annual vaccination is needed, respectively.

## Statistical analysis

Descriptive analysis of individual characteristics was analysed with vaccination rate. The association of each independent variable with the dependent variable were analysed with multivariable logistic regression analysis. All tests were 2-sided and p-values < 0.05 were considered significant.
